# General medicine, first-line medicine in Morocco: How is it perceived by medical students and how to enhance their interest in this career?

**DOI:** 10.4102/phcfm.v13i1.2837

**Published:** 2021-08-16

**Authors:** Maryam Fourtassi, Abda Naima, Yassamine Bentata

**Affiliations:** 1 Department of Physical Medicine and Rehabilitation, Faculté de Médecine et de Pharmacie d’Oujda, Mohammed Premier University, Oujda, Morocco; 2 Laboratory of Epidemiology, Clinical Research and Public Health, Faculté de Médecine et de Pharmacie d’Oujda, Mohammed Premier University, Oujda, Morocco; 3 Department of Nephrology, Faculté de Médecine et de Pharmacie d’Oujda, Mohammed Premier University, Oujda, Morocco

**Keywords:** general medicine, family medicine, medical studies, Morocco

## Abstract

In Morocco, family medicine does not exist, and it is general medicine that plays the role of family medicine and is also first line medicine and primary care. The current medical school curriculum is not fully in harmony with the real health needs of the population. We questioned 6th and 7th year students about the pertinence of two rotations in general medicine, that is, in a health centre and a private practice, via an anonymous questionnaire disseminated online in June 2020. A total of 266 responses were collected. Out of these, 41.5% of responses were enrolled in 6th year, versus 58.5% in 7th year. Then, 59.1% of students were females. In addition, 84.8% of them declared their intention to pursue a career in a medical specialty, whereas only 15.2% of them were interested in a career as a general practitioner. Notably, 67.4%, 26.5% and 6.1%, respectively, thought that general medicine was very undervalued, a little undervalued and not undervalued. It should be noted that 3.8%, 44.1% and 52.1%, respectively, were interested, somewhat interested and not at all interested in family medicine as a specialty if it was implemented. To that end, various actions need to be undertaken, including the introduction of quality teaching in the 6th and 7th years of medical studies, focused on the development of the knowledge and skills required, the strengthening of pre-existing practical training periods in public health and the introduction of a rotation in private practices of general medicine.

General practitioners (GPs) are considered as the first-line medical actors in most healthcare systems around the world, providing primary care to the population. The title of a GP in Morocco is granted to the medical school graduate upon completion of the seven years of medical studies, including all knowledge and skills-based examinations and public defence of a thesis in medicine (a work of research), in front of a jury of professors.^1^ The degree obtained is called a degree in medicine and the title of GP (or Doctor of Medicine), which is equivalent to that of a medical doctor (MD) in Anglophone countries, is conferred. The freshly graduated GPs can choose one of three possible paths; either join the public service and practice as a state-employed GP to be appointed in a public hospital or primary healthcare centre, start a private practice as a GP in an urban or rural area or take the Residency entrance examination, leading to a medical and or surgical specialty with the choice of specialty depending on his or her rank amongst accepted candidates. General practitioners retain the right to take the Residency entrance examination even after several years of general practice in the public or private sector.

In Morocco, and according to the latest official reports, GPs, who are the pillar of the national healthcare system, represent no more than 36% of the whole medical population, with 64% being specialised doctors.^2^ This quite unnatural pattern could be explained by the common undervaluation of this extremely important discipline amongst young medical graduates who would pursue a specialisation rather than start a practice as a GP. Indeed, the specialty of ‘Family Medicine’ does not exist yet in Morocco, and it is the GPs, or ‘non-specialised doctors’, who ensure all the medical care at the first level of our healthcare system, in both the public and private sectors, and in both urban and rural areas.

Aware of the importance of ‘Family Medicine’ in a health care system at a national level, Morocco has initiated a reflection on the implementation of a family medicine residency program. This project requires years of preparation, with substantial and specific goals to achieve such as the training of competent physician educators, the creation of family medicine departments, the identification and accreditation of internship locations and curriculum, the preparation and validation of training and practice-related legal texts, the drafting of competency reference standards and other requirements. However, this might not be enough to promote family medicine amongst young graduates who could still find it not that much attractive.

In order to investigate our nearly-graduates’ perception of general practice and family medicine (soon to be implemented), and their intention to pursue a career as a GP or family doctor, we conducted a survey using an anonymous online questionnaire that was sent, in June 2020, to students enrolled in the last two years of medical studies (6th and 7th year) in Moroccan medical schools. All participants gave their informed consent to take part in the survey, and their answers were analysed using Statistical Package for Social Sciences (SPSS) software. A total of 266 responses were collected. Out of these, 41.5% were enrolled in 6th year, versus 58.5% in 7th year. A total of 59.1% of students were females. In addition, 84.8% of them declared their intention to pursue a career in a medical specialty, whereas only 15.2% of them were interested in a career as a GP. Besides, 67.4%, 26.5% and 6.1%, respectively, thought that general medicine was very undervalued, a little undervalued and not undervalued. It was also noted that 3.8%, 44.1% and 52.1%, respectively, were interested, somewhat interested and not at all interested in family medicine as a specialty if it was implemented. When asked about their current general practice-oriented training and how it could be improved, 78.2% thought that it was necessary to have more practical training outside the university hospital. And, 65.8%, 23.3% and 10.9%, respectively, were very interested, somewhat interested and not interested in a general medicine rotation in a private practice setting. Also, 78.5% suggested a minimal duration of six weeks for this training period. The main ‘potential strength’ of this training, reported by the students, was the diversity of skills (professional, managerial and communication) that they could develop and or strengthen. The main ‘potential weakness’ of this training reported by the students concerned the quality of supervision and their apprehension about being unable to perform technical procedures or medical prescriptions.

In the light of these results, highlighting our students’ perceptions of their own current GP-oriented training, and pending the implementation on a national scale of ‘family medicine’ as a specialised medical discipline, we should orient the profile and training of the GP to that of a family doctor in order to better meet the population needs.

Currently, throughout their medical studies, students engage in clinical rotations only within public healthcare institutions, and mainly in hospital-based settings, where they are constantly exposed to patients requiring secondary or tertiary care. Health issues dealt with in primary care are rarely encountered, or almost never seen. Hence, young doctors who choose to practice as GPs, destined to be first-line doctors, find real difficulties in the field, and are often confronted to a kind of practice completely different from that of their training. The difficulties concern not only professional competencies, but also managerial and communication skills. Indeed, there are only two practical training periods in direct relationship with general medicine, taking place in a primary public healthcare centre; the first one usually scheduled in the 5th year of medical studies with a duration of 4 weeks, and the second in the 7th year of medical studies with a duration of 9 weeks. These short periods are not sufficient to provide young doctors with a sense of preparedness and self-confidence to practise general medicine. This is a main source of demotivation of young graduates from starting a general practice, along with other reasons like underpayment and social undervaluation of GPs, pushing most young doctors to choose the path of medical specialty, which opens the possibility of a more appealing and prestigious career. Indeed, the specialist doctor’s salary in the Moroccan public sector is 50% higher than the GP’s salary. The earning potential difference gets even wider in the private sector where the specialist interventions are highly remunerated compared to the ones performed by the GP. Also, the population tends to go directly consult a specialist in the private sector as there is no obligation to be referred by a GP first.

Three actions should be undertaken to better prepare medical students, and motivate them to choose the path of general medicine. The first action, consisting of the introduction of family medicine-related teaching modules in the 6th and 7th years of medical studies, has already been approved and accredited during the revision of the National Education Standards in 2015 and will be implemented by October 2020. These teaching modules are intended to consolidate the knowledge and skills acquired during the first five years of medical studies, and are mainly focused on children’s and women’s health, mental health, health of the elderly, chronic diseases, nutrition and ethics, all themes very frequently encountered in general medicine. The second action is to strengthen the rotations in general medicine in public healthcare institutions. An interesting idea would be to transfer the part-time 5th year rotation to a full-time 6th year rotation and lengthen the period from four weeks to eight weeks. This way, students will be given more responsibilities and will have more time to develop the necessary skills and to become more familiar with the professional environment of general medicine in the public sector. The third and last action should focus on introducing practical training periods in general medicine private practices. Like any educational activity, rotations in private practices require rigorous educational planning, including the definition of the training objectives in terms of professional, interpersonal, communication and managerial skills and the determination of adequate training assessment tools. In this context, the portfolio seems to be a good evaluation tool that leaves the learner with a certain degree of control, whilst allowing the evaluation of highly diverse skills. For this practical training to be successful, it will also be necessary to identify and accredit the participating medical practices, as well as to ensure the training of the supervising GPs.

Finally, we believe that the road towards the implementation of family medicine as an established specialty in Morocco should take place progressively, starting with the introduction of family medicine-related teaching modules in 6th and 7th years of medical studies, along with strengthening the pre-existing general medicine rotations in public healthcare institution and the introduction of a new clinical rotations in the private practice of general medicine, as a first significant step, towards a general medicine that is better adapted to the national context and more focused on the real national health needs. Then follows the second bigger step leading to the establishment of a family medicine that would be more solid, effective and successful.
